# Laser additive manufacturing of 3D meshes for optical applications

**DOI:** 10.1371/journal.pone.0192389

**Published:** 2018-02-07

**Authors:** Khamis Essa, Aydin Sabouri, Haider Butt, Fawzia Hamed Basuny, Mootaz Ghazy, Mahmoud Ahmed El-Sayed

**Affiliations:** 1 School of Mechanical Engineering, University of Birmingham, Birmingham, United Kingdom; 2 Indstry Service Complex, Arab Academy for Science and Technology and Maritime Transport, Abu Qir, Alexandria, Egypt; 3 Department of Industrial and Management Engineering, Arab Academy for Science and Technology and Maritime Transport, Abu Qir, Alexandria, Egypt; Beihang University, CHINA

## Abstract

Selective laser melting (SLM) is a widely used additive manufacturing process that can be used for printing of intricate three dimensional (3D) metallic structures. Here we demonstrate the fabrication of titanium alloy Ti–6Al–4V alloy based 3D meshes with nodally-connected diamond like unit cells, with lattice spacing varying from 400 to 1000 microns. A Concept Laser M2 system equipped with laser that has a wavelength of 1075 nm, a constant beam spot size of 50μm and maximum power of 400W was used to manufacture the 3D meshes. These meshes act as optical shutters / directional transmitters and display interesting optical properties. A detailed optical characterisation was carried out and it was found that these structures can be optimised to act as scalable rotational shutters with high efficiencies and as angle selective transmission screens for protection against unwanted and dangerous radiations. The efficiency of fabricated lattice structures can be increased by enlarging the meshing size.

## Introduction

In optics industry shutters are widely used to control the level of light exposure and also for optical switching/communication. A myriad of shutter designs and control procedures exist to suit the various optical applications and systems. This includes the large selection of shutters based on micro-mechanical rotators or micro-electro-mechanical systems (MEMs), which are available commercially [1,2]. On the other hand, Zhao et. al.[3] have demonstrated a different technique utilising microfluidics for producing large area and electrically tunable optical shutters. They utilised the phenomenon of dielectro wetting to switch the fluid layer (on top of a hydrophobic fluoropolymer) into droplets allowing the passage of light. The technology though interesting is not mature yet and needs further optimisation.

An extensive research has also been dedicated to produce liquid crystals (LCs) based optical shutters [4,5]. The advantage here is that LCs are electrically tunable. They present fast switching speeds and their materials properties can be easily tailored to suit the requirements of the optical systems. Moreover, the LC based shutters can also be tuned optically[6], where in the band gaps presented by the chiral nematic LCs can be tuned by an incident laser beam. Such a device is of potential importance for applications requiring remote switching involving the protection of sensor-based equipment from unwanted laser irradiation.

Here we present a tilt based shutter which allows light to pass only at certain angles of incidence. Such shutters can also be used as protective screens or layers to avoid unwanted irradiations, such as in the aeroplane pilot cabins which have recently been targeted by high powered ground lasers. The shutters were based on three dimensional metallic meshes made through the novel technique of additive manufacturing. Additive manufacturing (AM) techniques are a group of emerging technologies that have the ability to build 3D parts from bottom up by adding layer upon layer at a time. In this process, information of each layer is taken from a stereolithography (STL) file that is the CAD file sliced in approximated triangles and passed to a 3D printer [7–10]. Parts being built by 3D printing are becoming more popular in optics industry because it’s easy to build light weight components that are very durable [11–13]. Selective laser melting, also known as laser powder bed or 3D printing of metal, is a rapidly developing manufacturing technique that enables the fabrication of complex-shaped parts with intricate details. It involves an interaction of a laser beam with powder surface aiming towards achieving parts by melting and fusing of a series of powder layers on top of each other under an inert atmosphere according to a designed model. SLM is an adequate process for the fabrication of optics shutters because it uses laser spot size of 50–500 μm with layer thickness of 20–100 μm which resulted in as SLM part resolution of about 150 μm [14].

Ti6Al4V is a typical two-phase Ti alloy with a reputation of corrosion resistance, high specific strength. For this reason Ti6Al4V is called ‘‘space metal”, and has a wide application prospect in the military and civilian industry. Currently, Ti-6Al-4V is a widely common applied titanium alloy and its porous structure behaviours, including microstructure and the mechanical properties, have been studied extensively [15,16].

In this study we propose a novel Ti6Al4V shutter (directional transmitter) fabricated by selective laser melting which works based on the reflection of the light. Since this shutter is hollow, it has a very light weight and can be easily controlled using motors. In the following sections the method of the fabrication and the behaviour of the shutter are discussed.

## Fabrication

**Fig 1:**
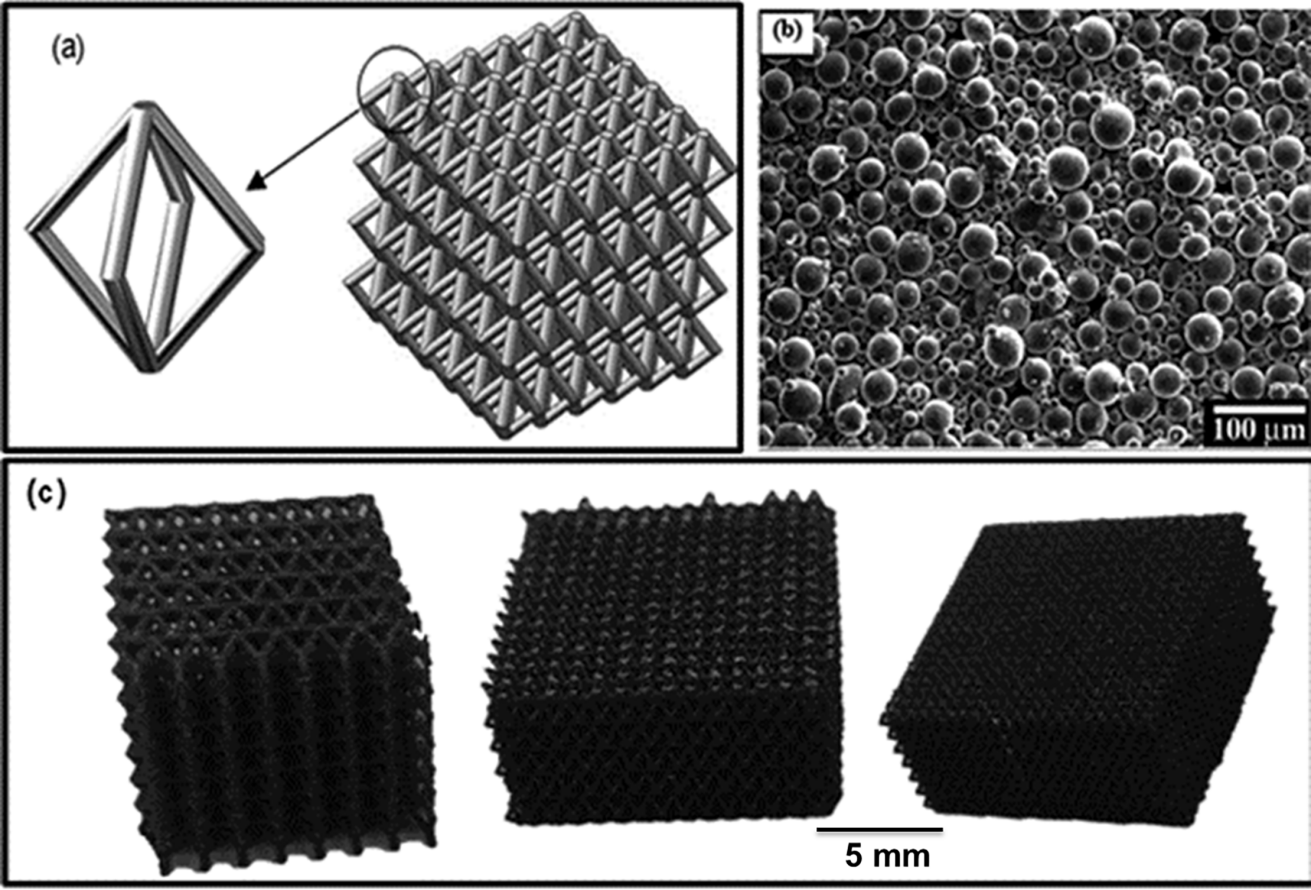
The lattice structures (a) The CAD design (b) An SEM image of Ti–6Al–4V powder morphology (c) As SLMed lattices with mesh sizes of 1 mm, 0.7 mm and 0.4 mm.

Lattice structures have a wide range of applications, such as biomedical implants, shock or vibration damping and acoustic absorption. In the current study they had been employed for the application of optics shutters where they were designed to produce tailored porous parts [6]. Selective laser melting was used as the proposed additive manufacturing (AM) tool for the fabrication of the proposed lattice structures. A 3D model of the lattice structure is shown in Fig 1(A). The lattice structures employed have diamond unit cell design with mesh length varying from 0.4 to 1mm. The unit cell shape was kept the same (i.e. nodally-connected diamond unit cells) as shown in Fig 1(A) but the strut size was varied to have lattice structures with different densities as shown in Fig 1(C). This was done based on the optimisation work reported by Qiu et al [17]. The powder used in the selective laser melting manufacturing of Ti–6Al–4V lattice structures was a gas atomised one supplied by TLS in the size range of 20–50 μm, Fig 1(B). As shown, most of the powder particles were generally spherical and few were irregularly-shaped particles which is a favourable morphology for the SLM. A Concept Laser M2 Cusing selective laser melting system, which uses an Nd:YAG (neodymium-doped yttrium aluminium garnet) laser with a wavelength of 1075 nm and a constant beam spot size of 50 μm with a melt pool of approximately of 150 μm in diameter, a maximum laser output power of 400 W and a maximum laser scanning speed of 7000 mm/s, was used to fabricate the lattice structures. To manufacture the Ti–6Al–4V, it is a must to avoid oxygen pick up by the power and molten metal and therefore the processing of the material was carried out under an argon atmosphere. The build process of SLM comprised of spreading a layer of the powder being developed over the build plate using a re-coater blade, scanning the predefined trajectory of the CAD model, and spreading another layer of the powder. The process was repeated until the component was built.

A set of laser process parameters with 200 W laser power, 1500 mm/s laser speed and 20 μm layer thicknesses were selected as a standard Ti–6Al–4V laser processing parameters. The process parameters were selected based on the extensive work done at the University of Birmingham [10,17]. Similar investigations were carried out to optimise lattice structures manufactured by SLM [18,19]. The lattice structures with three different mesh sizes are shown in Fig 1(C).

## Optical experiment

**Fig 2:**
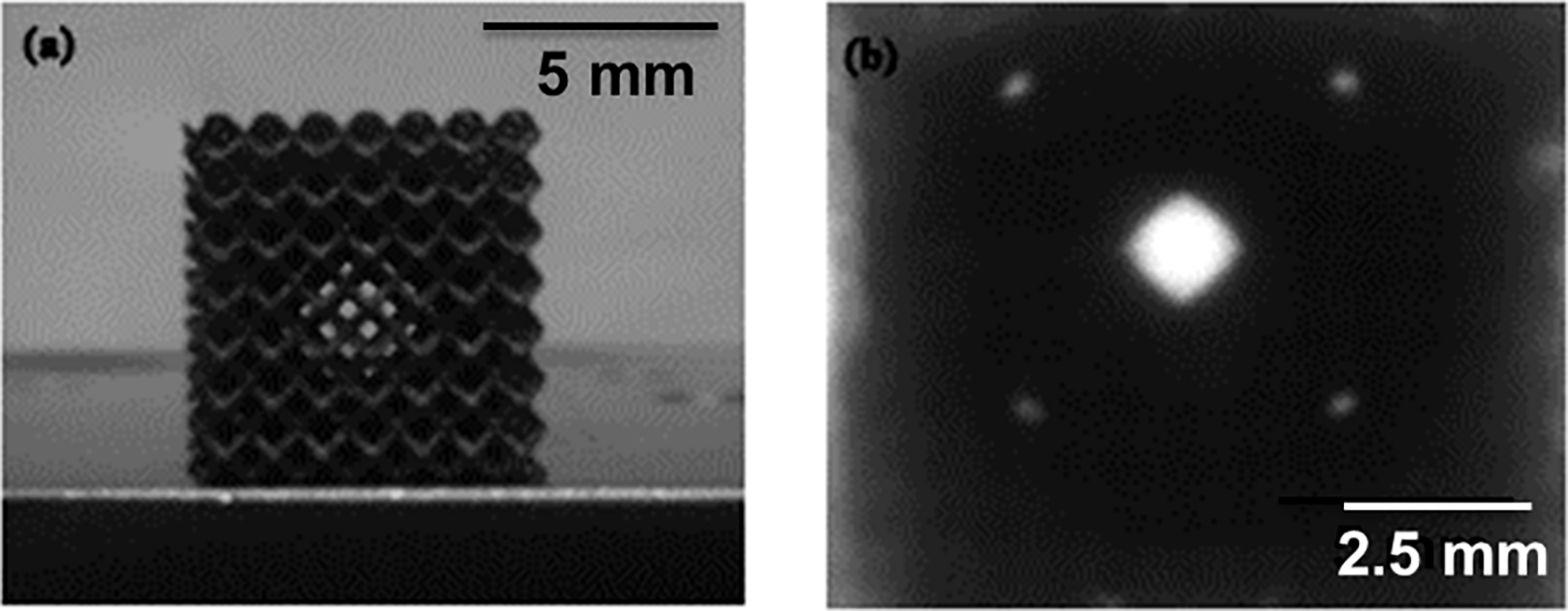
Image taken by camera showing the cube sample windows.

In order to achieve the characteristic of the optical shutter, a laser beam at different incident angles was shined on the samples and the power of transmitted light was measured by the power meter. It was observed that according to the geometry of the cubes, there are certain directions that are capable of transmitting the light (Fig 2). The shape of the transmitted light represented the respective shape of the mesh unit cells.

**Fig 3:**
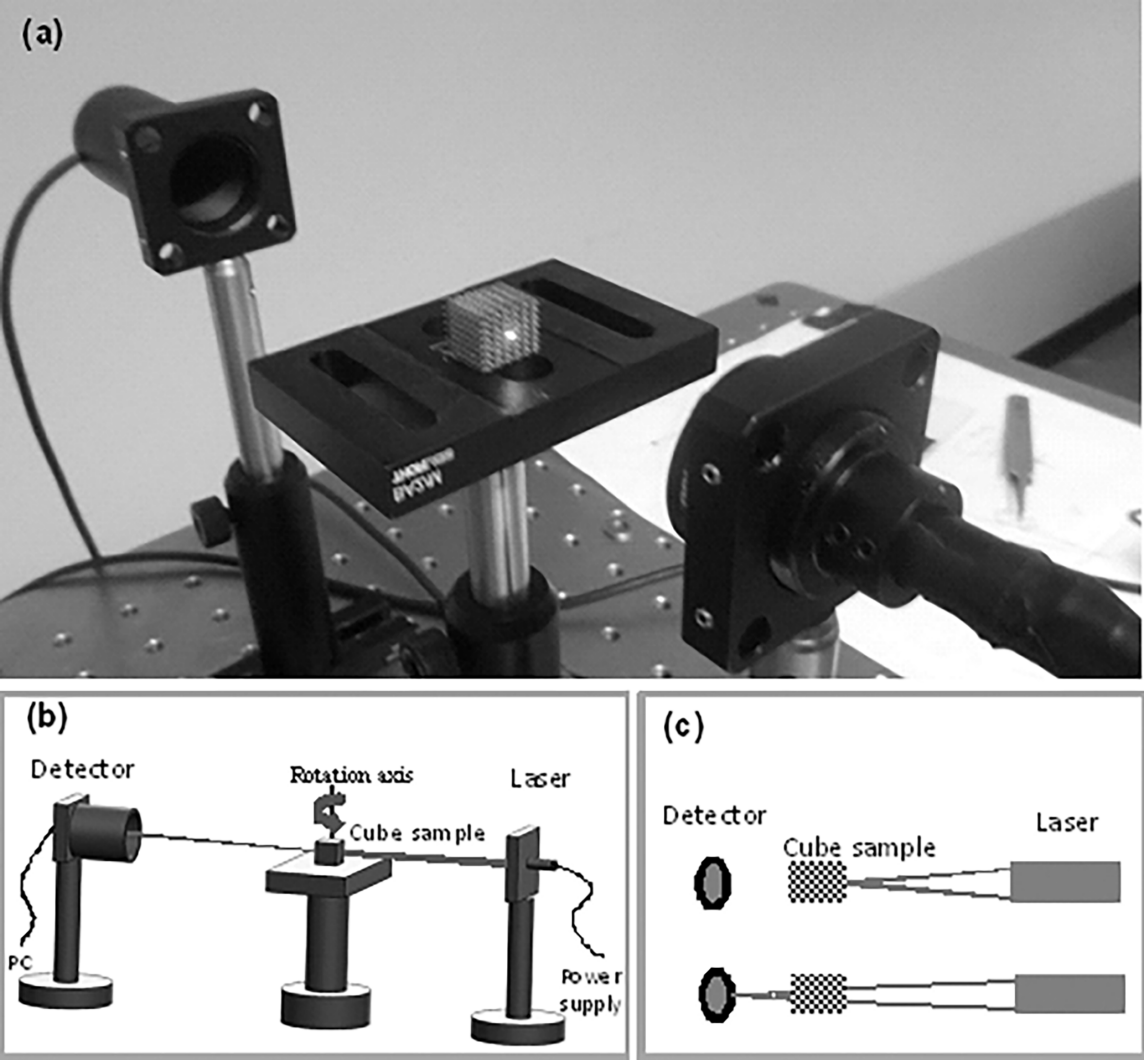
(a) Angular intensity measurement system. (b) Schematic view of the experimental setup. (c) The two scenarios studied with different focal point positions.

To investigate the characteristics of these windows, the sample was rotated 360 degrees with a step size of 1 degree along its vertical axis, while being illuminated by a red laser beam (with a wavelength of 650 nm and a power of 6 mW). On the other side of the sample a detector (optical power meter) was placed for measuring the power of the transmitted light. Fig 3(A) and 3(B) shows the experimental setup used for the measurements. The sample was placed on a motorised rotating stage controlled by computer software. The software also controlled the power meter and recorded the transmitted light reading for every rotation step with an error of about 5–10%. It was observed that the size of the laser spot had an influence on the behaviour of the cube samples. Therefore, these samples were studied under two conditions; the first where the beam was focused at the detector (i.e. larger spot size at sample) and the second condition where the spot was focused at the sample (Fig 3(C))

**Fig 4:**
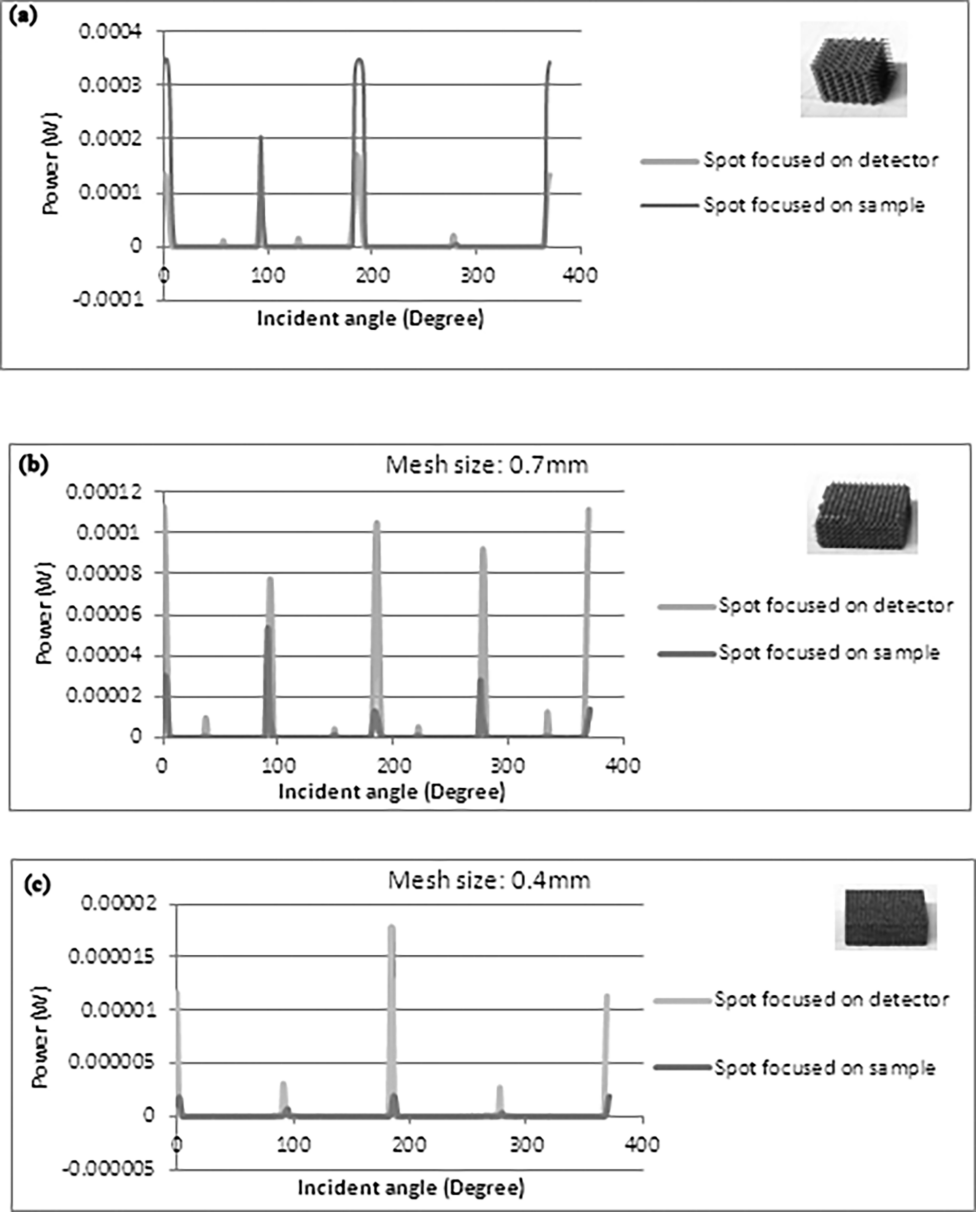
Measured angular transmission intensity for different mesh sizes (a) 1 mm (b) 0.7 mm and (c) 0.4 mm.

Fig 4 shows the measured optical intensity vs the incident angle. The samples displayed optical transmission not only when the light was normally incident but also at various other angles due to the existence of the optical windows produced by the 3D mesh geometries. This is of interest, that the meshes only transmit light at discrete angles and at all other angles the transmission is negligible (<0.1%). The angular intensity profiles revealed that there are more transmission peaks for the cases where the laser spot was focused at the detector. In this case the laser beam spot at the sample was larger and the light was more collimated to pass through the mesh windows. This also increased the chances of light passing through the additional paths. For the case of laser sport was focused at the sample the transmission was lower for all samples. This would be because the laser beam defocussed into the mesh, leading to a diffused beam that scattered within the mesh. As a result a lower power was transmitted normally through the window.

However, the intensities of the peaks showed different behaviours depending on the size of the meshes. For the samples with smaller mesh sizes, the intensity of peaks for larger spot sizes on the sample was greater than that associated with smaller spot sizes. This might be because of the higher reflection of the incident light at the surface of the sample while in larger spot sizes the chance of transmission was increased as more photons were penetrating into the cube. For the sample with the largest mesh size, smaller spots had fewer peaks but with higher intensities compared to larger spot sizes which was due to the reduced disturbance of the penetrated light inside the cube.

**Fig 5:**
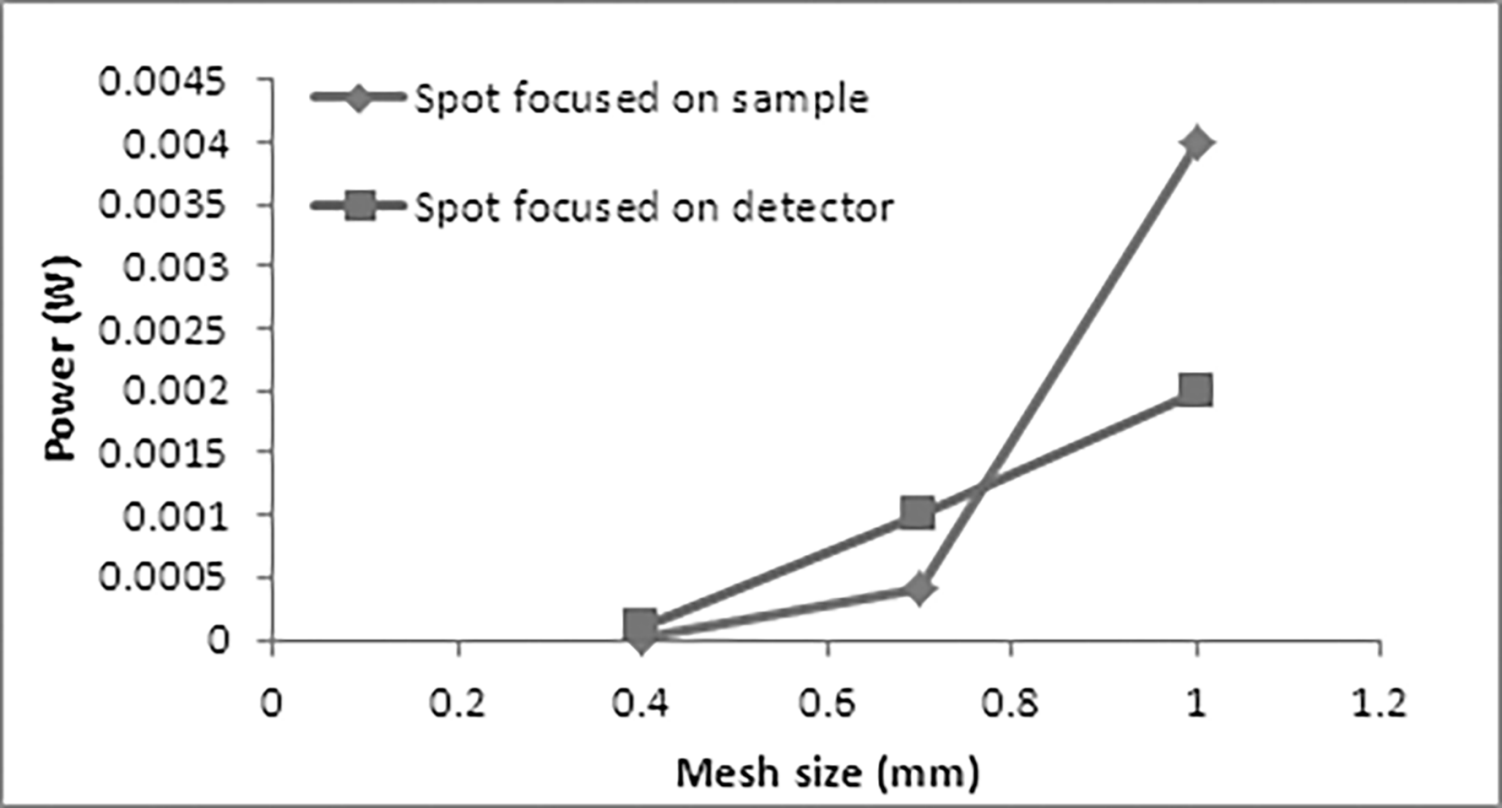
The efficiency of the sample cubes with different mesh sizes.

The area under achieved spectrum is a representative of the number of received photons by the detector for 360 degrees. The maximum peak could not be more than 6 mW which is the power of the emitted laser. In order to compare the efficiency of these samples for all incident angles, the integral of spectrum for each of them can be considered (Fig 5).

It can be seen that by reducing the mesh sizes, the transmission of the light was reduced for both cases. This is due to the increased blockage of the incident light for the samples with smaller mesh sizes at their surfaces.

## Conclusions

Selective laser melting technique of Ti–6Al–4V lattice samples was successfully used for the fabrication of optical shutters with different mesh sizes. It was shown that these cube samples can act as optical shutters or directional light transmitters. The controllable parameters on these structures were the spot size and the light incident angle. The efficiency of these shutters can be increased by enlarging the meshing size. Also, they have the advantage of being scalable due to their economical fabrication process. It was shown that the optical leakage for these shutters was very small (<0.1% for the sample with lowest efficiency).
